# Burden and risk factors of mental and substance use disorders among adolescents and young adults in Kenya: results from the Global Burden of Disease Study 2019

**DOI:** 10.1016/j.eclinm.2023.102328

**Published:** 2023-12-21

**Authors:** Kenya Adolescent Mental Health Group, Manasi Kumar, Simon Njuguna, Nabila Amin, Sarah Kanana, Albert Tele, Mercy Karanja, Nasri Omar, Obadia Yator, Christine Wambugu, David Bukusi, Marcia R. Weaver

**Keywords:** Mental disorders, Substance use, Adolescent mental health, Risk factors, Kenya

## Abstract

**Background:**

Mental and substance use disorders are a major public health concern globally, with high rates of disability, morbidity, and mortality associated with these. In low- and middle-income countries, such as Kenya, mental health is often given low priority, and resources for the prevention and treatment of mental and substance use disorders are limited. Adolescence and young adulthood are critical periods for the development of mental and substance use disorders, with many disorders emerging during this time. In Kenya, the burden and risk factors of mental and substance use disorders among adolescents and young adults is not well understood.

**Methods:**

The data used in this study were obtained from the Global Burden of Disease (GBD) Study 2019. We selected the data on the number of mental and substance use disorders among adolescents and young adults in Kenya from the GBD results tool. The data were extracted by mental health (MH) condition, by age group and by sex. We used descriptive statistical methods to summarise and present the data. Specifically, we calculated the disability-adjusted life-years (DALYs) rates, risk factors of mental and substance use disorders by age group and sex.

**Findings:**

In 2019, among 10–24-year-olds in Kenya, mental disorders ranked as the second leading cause of disability, following unintentional injuries, and accounted for 248,936 [95% uncertainty interval 175,033; 341,680] DALYs or 9.4% of 2,656,546 total DALYs. Substance use disorders accounted 15,022 [9948; 20,710] DALYs. Depressive, anxiety, and conduct disorders accounted for the most DALYs of mental disorders accounting for 3.1%, 2.3% and 1.7% of the total DALYs, respectively. The main risk factors for incident DALYs in 10–24-year-olds were bullying and victimization (66.5%). Childhood sexual abuse accounted for 13.7% of the DALYs, lead exposure accounted for 8.5% of the DALYs, intimate partner violence accounted for 11.3% of the DALYs (2%) with all victims being females, and illicit drug use accounted for (52.7%) of DALYs.

**Interpretation:**

Improved surveillance of mental health and substance use burden at national and county levels is needed. Focus on timely screening and intervention for idiopathic developmental intellectual disability, conduct disorder, and substance use disorder in young boys and depression, anxiety, and eating disorders in young girls and women is critically needed.

**Funding:**

MK is funded by 10.13039/100000061FIC/10.13039/100000025NIMH K43 TW 010716 and R33MH124149-03. The publication was made possible by funding from the Gates Foundation.

**Research in context**

**Evidence before this study**

Prior research on the burden and risk factors of mental and substance use disorders among adolescents and young adults in Kenya and Sub-Saharan Africa was limited. To gather existing evidence, we conducted a comprehensive literature search in databases including PubMed, Web of Science, Ovid, and Embase up until March 2023. Our search strategy used the following terms: ('mental disorders' OR 'substance use disorders' OR 'depressive disorders' OR 'anxiety disorders' OR 'conduct disorders' OR 'drug use disorders') AND ('adolescents' OR 'young adults' OR 'youth') AND ('Kenya' OR 'Sub-Saharan Africa') AND ('burden' OR 'prevalence' OR 'risk factors' OR 'epidemiology') AND ('Global Burden of Disease' OR 'GBD'). We also sought relevant publications from the World Health Organization (WHO) and other public health organizations. While previous studies have examined the global burden of mental disorders, there was a need for more specific regional and country-level data on the mental health of adolescents and young adults in Kenya, as well as a lack of population-based studies at the national level. This study aims to fill these knowledge gaps by presenting burden estimates and risk factors for mental and substance use disorders among 10–24-year-olds in Kenya, with a focus on gender, age groups, and specific disorders.

**Added value of this study**

The current study adds to the existing knowledge on the burden and risk factors of mental and substance use disorders among adolescents and young adults in Kenya. By using data from the Global Burden of Disease Study 2019, this study provides estimates of the prevalence of mental and substance use disorders at the national level. This information is crucial for policymakers and stakeholders in the mental health sector in Kenya to develop evidence-based strategies to address the burden of mental and substance use disorders.

**Implications of all the available evidence**

The findings of the current study have important implications for mental health policy and practice in Kenya. The current study also highlights the need for routine collection and reporting of mental health indicators at the national and subnational levels in Kenya, as recommended by the WHO Mental Health Action Plan 2013–2030.

## Introduction

Among the 25 leading causes of disability-adjusted life-years (DALYs) among people aged 10–24 years of age in 2019, depressive disorders ranked 4th, anxiety disorders ranked 6th, conduct disorders ranked 17th, and drug use disorders ranked 18th according to global burden of disease study data 1990–2019.^1^ Africa has the world's largest youth population, with over 60% of the population under the age of 25, according to the United Nations.^2^^,^^3^ Mental disorders account for a significant portion of disability due to non-communicable diseases. However, data on mental and substance use disorders is predominantly informed by high-income countries and is sparse for low-and middle-income countries (LMICs); the risk factors of mental and substance use disorders are also under-documented. LMICs account for 80% of the world’s population and yet very little empirical information exists on mental disorders from LMICs.^4^ This is particularly true for eastern Sub-Saharan Africa (SSA)—which accounts for a very low percentage of the population represented in mental disorder prevalence literature (0.1%–6.4%).^5^

The eastern SSA region has undergone a rapid epidemiological transition from communicable (malaria, HIV, tuberculosis) to non-communicable disease (NCD) burden (e.g., cardiovascular diseases, diabetes, musculoskeletal disorders). Kenya is one such country in eastern SSA that is undergoing such a transition–with improving life expectancy, lowered malnutrition, and a concomitant increase in burden of non-communicable diseases.^6^ Kenya is the largest economy in eastern SSA in terms of Gross Domestic Product (GDP) with a population of 47.5 million with nearly 60% of their population aged 24 or younger.^7^ Although much effort has historically been focused on lowering infectious disease burden, NCD burden is expected to increase in coming years particularly among adolescents and young adults.^8^

A serious gap in knowledge and evidence around burden and risk factors of mental health and substance use associated problems in SSA persists.^9^ A taskforce on mental health was launched by the Kenyan Ministry of Health in the year 2020 which noted that depression and anxiety disorders were the leading mental illnesses diagnosed in the country followed by substance use disorders. The report noted that among the different types of substances, alcohol contributes to the largest burden of substance use related illnesses in Kenya. Of great concern is how mental distress exacerbates high risk behaviors like alcohol abuse which is most prevalent in the 18–29-year-old age group.^10^ In another study carried out in Nairobi, Kamau et al. (2016) found that substance abuse was the most prevalent disorder amongst those who were seeking formal services followed by depression,^11^ with other sub-national studies highlighting a prevalence of 24% for alcohol use and 15% for substance use, respectively, in this population. Kamau et al. also found that the mean time to accessing care at the clinic after the onset of symptoms was 16.6 months, with the longest time taken to specialist care being 183 months.^11^ Despite the high burden of disease due to mental, neurological and substance disorders, there is considerable shortage of mental health specialists and neurologists.^12^ There are about 150 psychiatrists in Kenya. Most are based within Nairobi, the capital city. Outside of Nairobi, there is one psychiatrist per million population. There are 12 neurologists in Kenya and all practice primarily in the urban settings of Nairobi, Kisumu, and Mombasa and are primarily available in private settings.^10^

Although comprehensive reviews of the global burden of mental disorders have been reported previously, there is a need for a more detailed review of burden estimates by region and country. WHO’s Accelerated Action on Adolescent Health guidelines (AA-HA) (2017) recommend that action on improving health of adolescents start earlier given that half of all mental health disorders in adulthood start by age 14, but most cases are undetected and untreated.^13^ Adolescents and young adults face low health coverage, missing out on essential elements of a minimally sufficient health system. With 1.2 billion adolescents worldwide (defined by WHO as those aged 10–19 years), burgeoning mental health problems need to be addressed in time.^14^

Previous studies have indicated that mental and substance use disorders are a significant public health concern in Kenya. However, the available data on the burden and risk factors of mental and substance use disorders among adolescents and young adults in Kenya continue to be sparse. Furthermore, the national-level survey of non-communicable diseases in Kenya conducted using the World Health Organization STEPwise protocol did not include mental health.^15^ Thus, there is a lack of population-based studies of mental health at the national level in Kenya. Studies that have used WHO school health data to study African adolescents’ populations are pointing to greater burden and stress due to risk factors like social isolation, lack of parental and peer support and loneliness.

In this paper, we present burden estimates and risk factors for mental, and substance use disorders among 10–24-year-olds in Kenya by sex, age group, and disorder.

## Methods

### Study design

We report GBD 2019 results, which use all available sources of data to estimate the disease burden globally for 354 diseases and injuries for 204 countries and territories.^16^ Results were downloaded from the GBD Results tool. The mental disorders in the GBD framework include depressive disorders (major depressive disorder and dysthymia), anxiety disorders, bipolar disorder, schizophrenia, attention-deficit, and hyperactivity disorder (ADHD), autism spectrum disorders (ASDs), conduct disorder, idiopathic developmental intellectual disability (IDD), eating disorders, and personality disorders. Mental disorders were defined as per criteria in the Diagnostic and Statistical Manual of Mental disorder—fourth edition (DSM-IV)^17^ or the International Classification of Diseases (ICD-10). Substance use disorders were defined according to DSM-IV^17^ and ICD-10.^18^

The actual number of mental disorders was estimated with DisMod-MR (version 2.1), a Bayesian meta-regression framework that estimates non-fatal health outcomes by location, age, sex, and year. Years Lived with Disability (YLDs) were estimated by multiplying estimates at varying levels of severity and appropriate disability weights. Years of Live Lost (YLLs) for fatal causes were computed from observed deaths and reference standard life expectancy at the age of death, which was obtained from the GBD standard life table. Eating disorders were the only fatal mental disorder; both alcohol and drug use were fatal substance use disorders. All accessible data, including those for covariates, were used to develop a set of plausible models and eventually, the best ensemble predictive model to produce estimates of deaths and YLLs by location, age, sex, and year. DALYs, a summary measure of total health loss, were computed by adding YLLs and YLDs for each cause under mental and substance use disorders. We report 95% uncertainty intervals (UIs) for all estimates derived from the ordinal 25th and 975th draw of a total of 1000 draws of the posterior distribution at each step of the burden estimation process. More details on the epidemiological disease modeling process for mental and substance use disorders are available elsewhere.^19^

The GBD comparative risk assessment (CRA) framework was used to estimate exposure of risk factors associated with mental and substance use disorders and their attributable disease burden.^20^ The estimation of burden attributable to risk factors included ascertainment of risk–outcome pairs with sufficient evidence of a causal relation in the available literature. Meta-analyses were conducted of the relative risk of the outcome as a function of exposure to the risk. Exposure data for risk factors were used from all available data sources and strengthened with the incorporation of covariates for modelling. For each risk factor, the theoretical minimum risk exposure level was established as the lowest level of risk exposure below which the available evidence did not support a relationship between the risk and the disease outcome. For each risk factor, the population attributable fraction (PAF), of YLL and YLDs was computed using the distribution of exposures for each age, sex, location and year, and relative risk of each level of exposure.

### Data source

The data used in this study were obtained from the Global Burden of Disease (GBD) Study 2019, a comprehensive assessment of population health that uses a wide range of data sources to estimate the burden of disease and injury worldwide. The GBD study provides data on the prevalence, incidence, and mortality of various health conditions, including mental and substance use disorders, by age, sex, and location.

### Data selection

We selected the data on the prevalence and risk factors of mental and substance use disorders among adolescents and young adults in Kenya from the GBD study tool. The data were extracted by mental health (MH) condition, by age group (10–14, 15–19, and 20–24 years), and by sex (male and female).

### Data cleaning and preparation

The data were cleaned and prepared for analysis using the standard GBD procedures, which include removing outliers, imputing missing data, and adjusting for differences in data quality and completeness across locations and time periods. We also examined the data for any errors or inconsistencies and corrected them as needed.

### Data analysis

We used descriptive statistical methods to summarize and present the data. Specifically, we used the estimated numbers of mental and substance use disorders by age group and sex, and presented the results in tables and graphs.

### Ethical considerations

This study used publicly available data from the GBD study, which is ethically approved by the Institutional Review Board at the University of Washington. No personal identifying information was used in the analysis.

### Role of the funding source

The funders had no role in data collection, analysis, or interpretation of this manuscript. All authors confirm their participation in the shaping of the manuscript, had access to the data and accept responsibility for the decision to submit for publication.

## Results

**Fig. 1:**
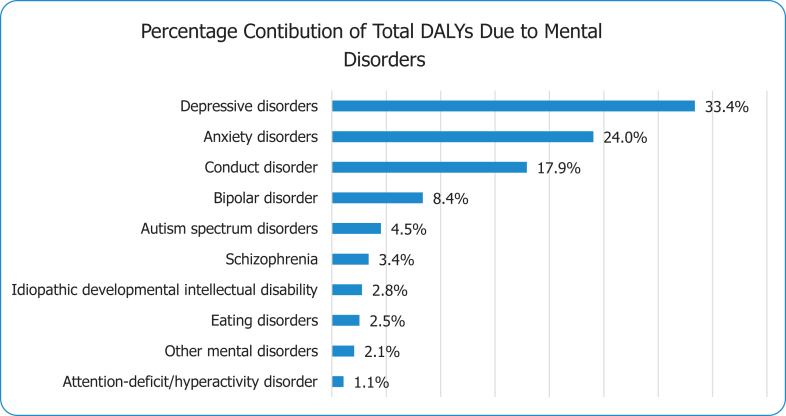
**Percentage of total DALYS from mental disorders by detailed (level 3)cause.**

**Fig. 2:**
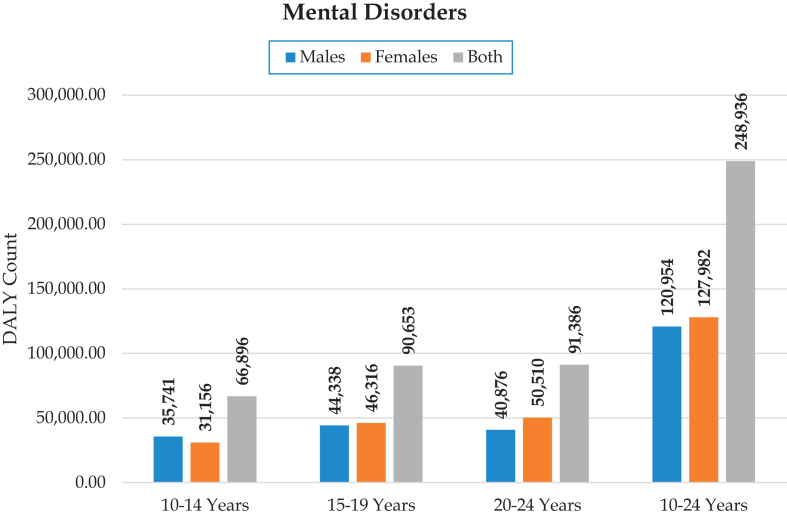
**DALYs from mental disorders bysex.**

In 2019, mental disorders were the 2nd leading cause of disability among 10–24-year-olds in Kenya accounting for 248,935 [95% uncertainty interval: 175,033; 341,680] DALYs of a total of 2,656,546 DALYs for both sexes. Depression contributed to 1/3rd (33.4%) of total DALYs followed by anxiety disorders (24.0%), conduct disorders (17.9%), bipolar disorder (8.4%), ASD (4.5%), schizophrenia (3.4%), IDD (2.8%), eating disorders (2.5%), ADHD (1.1%), others 2.1% [Fig. 1]. The total number of DALYs for females were higher than males [Table 1, Fig. 2 and Supplementary Figure S1]. Substance use disorders accounted for 15,022 [9948; 20,710] DALYs with slightly higher burden in males than females [Table 2 and Supplementary Figure S2]. Females had slightly higher overall DALYs attributed to mental disorders as compared to males in the 15–24 age group (127,982 [89,134; 174,990]) while males had slightly higher number compared to females in the 10–14 years (35,741 [23,978; 51,360]) out of a total of 66,896 [45,295; 96,575] in this age band.

**Table 1:** Number of mental disorders DALYs, with 95% UIs for Kenya, males, females and both sexes, disaggregated by age groups.

Type of mental disorder	Sub-group	Age category			Total
		10–14	15–19	20–24	10–24
**B.6: Mental disorders**	Male	35,740.5 [23,978.2; 51,359.9]	44,337.7 [30,577.6; 60,799.7]	40,876.1 [29,183.7; 55,256.9]	120,954.2 [84,596.0; 164,896.0]
	Female	31,155.9 [21,030.7; 44,733.1]	46,315.5 [31,770.3; 63,564.7]	50,510.2 [35,285.1; 68,724.7]	127,981.6 [89,134.4; 174,990.2]
	Both	66,896.4 [45,294.9; 96,574.9]	90,653.1 [62,092.7; 124,606.2]	91,386.3 [64,606.6; 123,800.0]	248,935.8 [175,033.1; 341,679.9]
B.6.1: Schizophrenia	Male	168.3 [85.5; 276.2]	1149.1 [686.0; 1737.9]	3110.3 [1943.5; 4595.2]	4427.7 [2832.0; 6468.0]
	Female	143.0 [71.5; 237.1]	992.9 [592.5; 1503.6]	2845.0 [1791.1; 4236.7]	3980.9 [2574.2; 5815.3]
	Both	311.2 [158.0; 513.4]	2142.0 [1288.1; 3234.2]	5955.3 [3757.1; 8796.3]	8408.6 [5422.6; 12,394.9]
B.6.2: Depressive disorders	Male	4246.0 [2454.4; 7058.2]	12,849.9 [8098.2; 19,082.9]	17,225.9 [10,828.5; 25,769.9]	34,321.8 [21,790.5; 50,533.8]
	Female	6020.2 [3421.2; 9953.6]	18,116.3 [11,443.6; 26,801.5]	24,627.3 [15,647.3; 36,908.1]	48,763.8 [31,503.7; 71,186.1]
	Both	10,266.2 [5886.5; 16,784.9]	30,966.2 [19,627.2; 46,079.0]	41,853.2 [26,538.3; 62,142.7]	83,085.6 [53,554.3; 121,530.2]
B.6.2.1: Major depressive disorder	Male	3518.8 [1908.9; 6154.8]	10,789.5 [6539.8; 16,708.4]	14,223.4 [8455.0; 21,933.9]	28,531.7 [17,417.7; 42,825.4]
	Female	4926.3 [2639.2; 8366.4]	14,794.7 [8908.3; 22,496.0]	19,596.8 [11,245.8; 30,303.0]	39,317.8 [24,452.1; 59,450.1]
	Both	8445.1 [4537.1; 14,444.7]	25,584.2 [15,376.3; 39,053.6]	33,820.2 [19,976.4; 51,780.0]	67,849.5 [42,076.8; 101,777.8]
B.6.2.2: Dysthymia	Male	727.2 [367.1; 1265.1]	2060.5 [1162.7; 3325.6]	3002.5 [1740.3; 4744.9]	5790.1 [3468.5; 9050.9]
	Female	1093.9 [557.8; 1868.8]	3321.6 [1888.4; 5372.3]	5030.5 [2928.9; 7880.2]	9446.0 [5606.6; 14,510.1]
	Both	1821.0 [935.4; 3121.4]	5382.1 [3047.8; 8536.5]	8033.0 [4741.7; 12,588.3]	15,236.1 [9110.7; 23,503.1]
B.6.3: Bipolar disorder	Male	1249.6 [610.5; 2069.8]	4260.6 [2228.4; 6836.2]	4775.3 [2659.4; 7634.3]	10,285.5 [5637.9; 16,254.4]
	Female	1256.7 [631.2; 2076.6]	4308.9 [2251.8; 6857.5]	4945.0 [2718.5; 8072.3]	10,510.6 [5743.4; 16,489.4]
	Both	2506.3 [1245.9; 4174.8]	8569.5 [4480.2; 13,620.6]	9720.3 [5335.0; 15,628.6]	20,796.1 [11,459.8; 32,642.6]
B.6.4: Anxiety disorders	Male	6753.1 [4166.9; 10,201.3]	9211.6 [6145.3; 13,263.5]	9215.5 [5770.4; 13,735.5]	25,180.2 [16,843.0; 36,080.5]
	Female	8943.1 [5603.4; 13,431.8]	12,556.6 [8362.2; 17,981.3]	13,171.3 [8238.8; 19,510.9]	34,671.0 [23,156.6; 49,627.2]
	Both	15,696.2 [9921.0; 23,569.6]	21,768.2 [14,444.2; 31,234.1]	22,386.8 [14,054.4; 33,215.2]	59,851.2 [40,268.9; 85,669.7]
B.6.5: Eating disorders	Male	413.0 [202.0; 690.9]	964.8 [503.3; 1703.6]	973.7 [489.5; 1731.2]	2351.5 [1356.1; 3710.8]
	Female	591.5 [319.2; 954.6]	1620.7 [854.8; 2739.6]	1745.5 [964.8; 2890.8]	3957.7 [2282.4; 6183.6]
	Both	1004.5 [524.6; 1645.6]	2585.5 [1366.2; 4414.9]	2719.2 [1457.0; 4613.1]	6309.2 [3662.5; 9924.7]
B.6.5.1: Anorexia nervosa	Male	162.2 [77.3; 280.5]	332.6 [173.9; 600.4]	251.6 [130.9; 448.3]	746.3 [393.5; 1309.4]
	Female	352.1 [177.9; 578.3]	835.6 [439.3; 1497.7]	702.9 [380.6; 1272.1]	1890.6 [1020.2; 3269.4]
	Both	514.3 [261.0; 842.5]	1168.2 [618.2; 2069.8]	954.5 [508.3; 1710.4]	2636.9 [1426.2; 4598.0]
B.6.5.2: Bulimia nervosa	Male	250.8 [94.1; 503.8]	632.2 [278.0; 1269.5]	722.2 [293.6; 1416.0]	1605.2 [830.0; 2738.2]
	Female	239.4 [88.2; 474.0]	785.1 [345.0; 1633.0]	1042.6 [464.7; 1979.8]	2067.1 [1038.9; 3646.0]
	Both	490.2 [193.5; 980.2]	1417.3 [626.8; 2888.3]	1764.8 [766.0; 3449.2]	3672.3 [1878.6; 6302.8]
B.6.6: Autism spectrum disorders	Male	3175.3 [2075.4; 4569.9]	2813.4 [1829.8; 4076.1]	2244.6 [1468.6; 3221.4]	8233.3 [5367.2; 11,860.0]
	Female	1138.0 [740.6; 1674.6]	1014.7 [656.4; 1497.8]	840.4 [547.4; 1242.7]	2993.2 [1947.6; 4392.8]
	Both	4313.3 [2811.1; 6306.9]	3828.2 [2486.3; 5528.3]	3085.0 [2009.6; 4444.8]	11,226.5 [7321.8; 16,271.6]
B.6.7: Attention-deficit/hyperactivity disorder	Male	900.7 [475.6; 1609.3]	664.6 [353.1; 1181.8]	377.8 [202.5; 682.7]	1943.1 [1052.0; 3431.5]
	Female	323.3 [166.5; 580.9]	242.4 [127.5; 434.5]	144.0 [75.2; 258.8]	709.7 [378.8; 1263.8]
	Both	1224.0 [650.3; 2201.8]	907.0 [482.9; 1593.9]	521.8 [279.8; 948.5]	2652.8 [1441.0; 4718.3]
B.6.8: Conduct disorder	Male	17,236.2 [9757.2; 27,574.0]	10,204.5 [5559.2; 16,561.3]	0.0 [0.0; 0.0]	27,440.7 [15,598.6; 43,394.8]
	Female	11,409.6 [6078.0; 18,919.8]	5766.5 [2940.3; 9696.8]	0.0 [0.0; 0.0]	17,176.1 [9163.9; 28,184.4]
	Both	28,645.8 [16,041.0; 46,132.1]	15,971.0 [8500.9; 25,885.0]	0.0 [0.0; 0.0]	44,616.8 [25,343.8; 70,764.8]
B.6.9: Idiopathic developmental intellectual disability	Male	1441.9 [643.9; 2508.3]	1269.0 [543.7; 2193.8]	972.2 [417.7; 1674.4]	3683.0 [1612.1; 6369.8]
	Female	1232.0 [565.5; 2119.6]	1092.6 [500.2; 1886.3]	874.8 [394.0; 1513.8]	3199.5 [1457.6; 5498.1]
	Both	2673.9 [1205.4; 4604.7]	2361.6 [1059.1; 4047.2]	1847.1 [815.6; 3191.2]	6882.5 [3072.2; 11,822.1]
B.6.10: Other mental disorders	Male	156.5 [78.8; 267.9]	950.1 [489.9; 1625.4]	1980.7 [1051.5; 3297.0]	3087.3 [1625.2; 5185.2]
	Female	98.5 [46.1; 175.7]	603.8 [273.5; 1052.4]	1316.9 [660.6; 2249.1]	2019.2 [986.1; 3504.5]
	Both	254.9 [127.8; 441.7]	1554.0 [770.1; 2657.1]	3297.6 [1740.2; 5535.9]	5106.5 [2638.9; 8647.5]

**Table 2:** Number of Substance use disorders, with 95% UIs for Kenya, males, females and both sexes, disaggregated by age groups.

Type of drug/substance	Sub-group	Age category			Total
		10–14	15–19	20–24	10–24
**B.7: Substance use disorders**	Male	172.6 [86.4; 292.4]	2439.5 [1631.9; 3406.4]	5927.9 [3992.1; 8188.2]	8540.1 [5758.1; 11,623.5]
	Female	126.2 [68.5; 205.6]	1717.1 [1119.4; 2434.7]	4639.0 [2927.5; 6574.6]	6482.3 [4179.3; 9114.9]
	Both	298.8 [157.9; 495.4]	4156.6 [2764.9; 5839.9]	10,566.9 [6888.6; 14,659.8]	15,022.4 [9947.9; 20,709.8]
B.7.1: Alcohol use disorders	Male	130.8 [69.9; 220.6]	1064.7 [626.3; 1674.8]	2438.1 [1469.5; 3864.7]	3633.6 [2239.5; 5609.3]
	Female	107.9 [56.4; 183.7]	846.1 [495.8; 1327.8]	1965.0 [1147.6; 3146.5]	2919.1 [1775.5; 4557.1]
	Both	238.8 [128.0; 407.0]	1910.8 [1121.2; 2999.8]	4403.1 [2621.2; 6965.6]	6552.7 [4022.6; 10,208.9]
B.7.2: Drug use disorders	Male	41.8 [3.3; 105.1]	1374.9 [915.3; 2033.2]	3489.8 [2271.1; 4991.4]	4906.5 [3271.1; 6943.9]
	Female	18.3 [1.9; 46.9]	871.0 [542.4; 1356.0]	2673.9 [1635.1; 4130.2]	3563.2 [2188.4; 5502.9]
	Both	60.1 [5.3; 151.9]	2245.9 [1460.3; 3345.0]	6163.7 [3910.6; 9025.6]	8469.7 [5416.6; 12,371.9]
B.7.2.1: Opioid use disorders	Male	0.0 [0.0; 0.0]	477.0 [292.7; 772.5]	1534.6 [900.2; 2548.9]	2011.6 [1198.2; 3276.7]
	Female	0.0 [0.0; 0.0]	469.2 [231.0; 829.4]	1782.9 [900.0; 3144.4]	2252.1 [1155.7; 3974.1]
	Both	0.0 [0.0; 0.0]	946.2 [543.0; 1590.2]	3317.5 [1784.0; 5708.5]	4263.7 [2320.4; 7286.0]
B.7.2.2: Cocaine use disorders	Male	0.5 [0.0; 1.5]	37.5 [18.4; 67.8]	68.1 [39.1; 113.8]	106.2 [61.5; 176.0]
	Female	0.3 [0.0; 0.9]	16.0 [6.6; 33.1]	20.1 [9.3; 38.4]	36.4 [16.8; 66.7]
	Both	0.9 [0.1; 2.3]	53.5 [26.9; 100.6]	88.3 [50.7; 148.9]	142.6 [81.0; 236.9]
B.7.2.3: Amphetamine use disorders	Male	0.0 [0.0; 0.0]	176.9 [78.2; 314.7]	723.3 [346.0; 1268.6]	900.2 [429.5; 1577.1]
	Female	0.0 [0.0; 0.0]	88.0 [39.6; 153.1]	354.1 [159.0; 624.3]	442.1 [200.6; 774.6]
	Both	0.0 [0.0; 0.0]	264.8 [121.5; 467.7]	1077.4 [517.2; 1870.6]	1342.3 [643.9; 2329.3]
B.7.2.4: Cannabis use disorders	Male	40.9 [2.4; 104.4]	499.5 [181.6; 1041.7]	569.9 [283.9; 1004.9]	1110.3 [522.0; 2043.9]
	Female	17.7 [1.5; 46.5]	214.8 [80.6; 447.2]	251.1 [122.3; 445.4]	483.7 [234.0; 888.8]
	Both	58.6 [3.9; 149.8]	714.3 [265.9; 1473.8]	821.0 [409.8; 1457.4]	1593.9 [754.8; 2919.5]
B.7.2.5: Other drug use disorders	Male	0.4 [0.0; 1.0]	184.0 [112.9; 279.6]	593.9 [347.8; 967.9]	778.2 [472.4; 1234.0]
	Female	0.2 [0.0; 0.6]	83.1 [48.8; 130.4]	265.7 [135.9; 444.9]	348.9 [186.5; 572.3]
	Both	0.6 [0.1; 1.5]	267.1 [167.2; 411.2]	859.5 [486.6; 1389.7]	1127.2 [661.4; 1801.6]

**Fig. 3:**
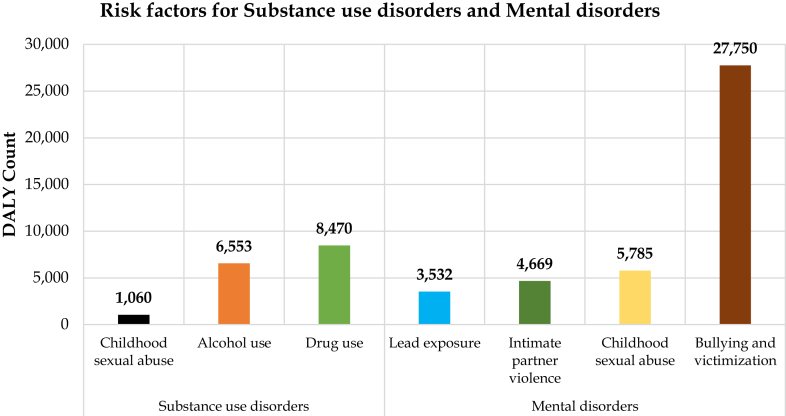
**Risk factors for mental and substance usedisorders.**

Among the risk factors that were associated with mental disorders, bullying and victimization accounted for 2/3rd (66.5%) of all the DALYs attributed to risk factors [Fig. 3 and Supplementary Table S1]. Among the 27,749 DALYs associated with bullying and victimization, females have a higher proportion (56.7%) than males (43.3%). Childhood sexual abuse accounted for 13.7% of the DALYs, with higher proportion of females (68.5%) than males (31.5%). Intimate partner violence accounted for 11.3% of the DALYs all of them being females (100.0%). Lead exposure accounted for 8.5% of the DALYs with higher proportion of them being males (56.2% vs 43.8%) (Supplementary Table S1). Among the risk factors that were associated with substance use disorders more than half (52.7%) were attributed to drug use (8470 [5417; 12,372]). Among these 57.9% were males and 42.1% were females (see Supplementary Table S2). Alcohol use accounted for 40.7% of DALYs with more than half (55.5%) being males. Childhood sexual abuse accounted for accounted for 6.6% of the DALYs, with 52.5% identified in females (Supplementary Table S2).

## Discussion

Depressive and anxiety disorders were the most prevalent mental disorders among 10–24-year-olds in Kenya. The number of DALYs for depressive disorders, anxiety disorders, bipolar disorder, and eating disorders were higher for females compared to males. Whereas the number of DALYs for schizophrenia, conduct disorder, autism spectrum disorder, ADHD, IDD, personality disorders and substance use disorders were higher for males compared to females. The patterns of high burden associated with anxiety, depressive and conduct disorders are consistent with other studies.^21^^,^^22^ The increased burden of internalizing disorders overall and especially in females is also consistent with the other studies.^11^^,^^23^ The DALYs for depressive and anxiety disorders was higher for older age groups. This may be attributed to known changes in stressors that accompany transitions in older age groups. Whereas the DALYs rate for conduct disorders and ADHD are lower for older age groups. These disorders may manifest in a different set of social and functional impairments and are also classified as different diagnoses as one reaches adulthood.

The findings above present an informative disease burden profile of mental and substance use disorders in Kenya among children and young adults ages 10–24. This age group is encounters important transitions and mental disorder burden may lower long-term productivity and potentially be associated with the onset of other comorbid disorders. Globally, populations between the ages of 10–24 are generally known to have low mortality rates and very good physical health with relatively low utilization of health services.^24^ However, the evidence on the burden of mental disorders in this age group in Kenya supports the need for development of services for this population.^25^ Even though the burden is higher in 15–24 years old we would like to underscore the importance of service set up for adolescents ages 10–14 years old—they need both treatment, and prevention and promotive mental health services.

The findings presented here highlight the need for preventive services to address the burden of mental disorders in this age group in Kenya. This would however need significant boosts in funding allocated to these efforts. In 2019, development assistance for health (DAH) for non-communicable diseases (which includes mental health) for SDG 3 targets was $0.7 billion for 135 lower and middle income countries—which is less than 2% of the total estimated DAH in 2019.^26^ Global health financing has historically been prioritised for malaria, HIV/AIDs, and tuberculosis—which are some of the leading causes of disability and mortality in much of SSA. However, it is important to align funding priorities with epidemiological shifts in LMICs that are likely to be accompanied by an increase in non-communicable disease burden including mental disorders.

Early intervention is critical to address growing numbers of depressive and anxiety disorders, eating disorders in females and rising substance use in adolescents.^9^ Historically, African mental health specialists have not paid attention to eating disorders due to a belief that a high level of individuation and prosperity was associated with these disorders. However, the rise in eating disorders in females points to issues around family dynamics, self-esteem, and body image that appear to contribute to rise in eating disorders. Studies in this area point to a poor quality of life satisfaction in female adolescents than their male counterparts.^27^^,^^28^ However there are also reports on rise of eating disorders in youth due to changes in quality of life, and urban westernized life style impacts on parent-child relationships and parenting.^29^ There is evidence that HIV cases in highly emaciated youth may be presenting themselves with HIV associated anorexia that can also be classified under eating disorders.

The burden of mental disorders poses a particular challenge for female adolescents. Depression among girls is known to be associated with sexual abuse, unplanned sexual encounters leading to adolescent pregnancy as well as HIV exposure.^30^ We also noted a significant burden associated with substance use in girls. Sexual and reproductive health programs integrated with mental health and substance use literacy, prevention and promotion of behavioral health targeting adolescent girls are needed. School based interventions including universal programs targeting teachers would be particularly important for early identification, screening and management of mental distress and disorders.^31^

It is also important to account for an increase in other mental disorders and psychoses/schizophrenia over time.^22^ The overlap between developmental disability and psychopathology is high in Kenyan adolescents. ADHD and other pervasive developmental disorders are known to be risk factors for development of psychoses. Early screening of psychoses in health facilities as well as schools and communities would be important. Subnational data are needed to better tailor intervention strategies and delivery of services. We will need to collect more data to support improved subnational estimates in the future.

Developmentally and culturally validated assessment and screening tools for key illnesses are also critical.^32^ The missing data is also attributable to lack of culturally validated clinical assessments at specialist and primary care facilities as well as paucity of robust public health monitoring of adolescent mental health in LMICs. The WHO Child and Adolescent Mental Health Atlas^33^ underscored that there were multiple factors at play—lack of knowledge, investment, financing, poor training of child and adolescent mental health professionals in SSA.

Risk factors like bullying victimization, childhood sexual abuse and intimate partner violence are noted to be significantly impacting burden of mental disorders in children and adolescents.^34^ Both relate to broader issues of negative social determinants of child and adolescent mental health and adverse childhood experiences which contribute towards psychopathology development in adolescence and adulthood. Awareness campaigns are needed in schools, communities, and in formal institutions around prevention of bullying and childhood sexual abuse. Pathways of proper and timely management of these risks is equally important. It was reported that annually around 98.2 million IQ points are lost in Africa due to lead exposure, translating into economic losses of US$ 134.7 billion.^35^ The detrimental health, educational and social impacts need serious public health and public policy level interventions. Lead exposure in drinking water, food storage and packing, toys, cosmetics, lead acid batteries, house paints etc. need to be regulated. Urban children are most at risk and both the low and high levels of lead exposure present significant psychiatric and neurodevelopment morbidity and mortality (with symptoms of hyperactivity, hearing loss, reading and learning disabilities, anemia, brain, liver, kidney, nerve and stomach damage, convulsions etc.).^36^

Previous study on Kenya using GBD data reported that though health disparities were high, and data on mortality and morbidity in Kenya continues to be sparse, there is a need to meaningfully connect existing data to create a holistic health profile.^6^ There are other studies that have recommended that GBD has missed out on sub-national data and precision public health approaches have been advanced.^37^ Another paper reporting on findings of Kenya NCDI (non-communicable diseases and injuries) poverty commission in 2020 found that the majority of disability-adjusted life-years occurred before age of 40 and that the poorest wealth quintiles experience not only considerably higher deaths rate from NCDIs but there was poorer diagnosis and treatment and availability of NCDI-related health services.^38^ Similarly our findings also imply that treatment services for adolescents and young people in need of care is the most urgent concern. For the risk factors we see for substance use and mental disorders we also need timely and culturally appropriate diagnostic measures.

Kenyan National Mental Health Plan 2021–2025 now provides a framework for the county and national government to start making progress in timely assessment, management including preventive and promotive care for young populations in Kenya.^39^ The 2021 WHO’s mental health investment case for Kenya provided useful pointers to treatment, prevention and promotion service development within the country. It also identified that the intervention packages for scaled-up treatment of depression, anxiety and alcohol use disorder (some of the commonest mental health conditions in Kenya) would have a high return on investment (ROI), resulting over 20 years in 5.1, 3.8 and 2.7 KES, respectively, for every 1 KES invested. Many of these intervention packages are available for scale up now and with these ROI figures the improved investment in youth mental health will likely yield great results.

UN Sustainable Development Goals (SDGs) 3 and 4 which target ‘good health and well-being’ and ‘inclusive equitable quality education and life-long learning opportunities’ underline the importance of provision of essential health services including mental health, substance use and suicide prevention programs. UN SDG target 3 indicators 3.4 and 3.5 aim to reduce by one third premature mortality from non-communicable diseases through prevention and treatment and promote mental health and well-being by 2030 and provide full range of services that entail treatment, rehabilitative, prevention and promotion activities strengthening mental health.

One limitation of this analysis is that we were unable to quantify co-occurring disorders as GBD data does not currently allow us to track multimorbidity. It is important to consider the increase in disability resulting from co-occurring physical and mental disorders as well as the overlap in risk factors that could exacerbate these conditions.^40^ Another limitation is that subnational data from the provinces of Kenya are sparse and therefore detailed subnational assessment of mental disorder burden in Kenya are primarily extrapolations of national results. This is an area for future development. We did not do primary data collection in mental disorders where there is gap in knowledge about epidemiology, their clinical presentation and associated risk factors. The data presents core findings of GBD 2019 papers that have been published earlier.

In conclusion, Kenya is a young nation, and the mental and physical well-being of its young population is important. Mental disorders were the 2nd leading cause of disability among 10–24 years old in Kenya with girls. Age and gender differentiated intervention and outreach strategies would be needed to mitigate impact of these disorders. The risk factors associated with mental disorders like childhood sexual abuse, intimate partner violence and bullying victimization need to be addressed through gender responsive school, family level and community-based sensitization and interventions. Depressive disorders, anxiety disorders, alcohol use disorders accounted for the highest prevalence followed by conduct disorders, bipolar disorders and these conditions need prioritization. Early screening and interventions for neurodevelopmental and pervasive development disorders should address risk factors like lead poisoning.

The findings of the current study have important implications for mental health policy and practice in Kenya. The Lancet Commission on Global Mental Health and Sustainable Development called for countries to fill gaps in knowledge regarding mental health, and the current study addresses this call. Furthermore, the study provides data that can inform the development of policies and interventions to address the burden of mental and substance use disorders among adolescents and young adults in Kenya.^41^ The current study also highlights the need for routine data collection and reporting of mental health indicators at the national level in Kenya, as recommended by the WHO Mental Health Action Plan 2013–2030.^42^ The report by WHO in October 2021,^43^ which highlights the global shortfall in investment in mental health, underscores the importance of studies such as the current one to raise awareness and provide data to inform mental health policy and practice.

## Contributors

MK developed the protocol with MW after consultations with IHME team. MW extracted the data and AT worked on creating summaries of the result tables. Results and discussion were jointly developed in consultation with SN, NA, SK, OY, MK and MK, SN, NA, SK, AT, MK, NO, OY, CW, DB, & MW all read, commented and approved the work for submission. AT, MK and MW have verified the data collected here. All authors confirm that they had full access to all the data in the study and accept responsibility for the decision to submit for publication.

Please see Appendix for more detailed information about individual author contributions to the research, divided into the following categories: managing the overall research enterprise; writing the first draft of the manuscript; primary responsibility for applying analytical methods to produce estimates; primary responsibility for seeking, cataloguing, extracting, or cleaning data; designing or coding figures and tables; providing data or critical feedback on data sources; developing methods or computational machinery; providing critical feedback on methods or results; drafting the manuscript or revising it critically for important intellectual content; and managing the estimation or publications process.

## Data sharing statement

All data shared here is available open-access through GBD [https://www.healthdata.org/research-analysis/gbd].

## Declaration of interests

Authors report no conflict of interest.
