# Supplementary material for: Burden and risk factors of mental and substance use disorders among adolescents and young adults in Kenya: results from the Global Burden of Disease Study 2019
Source: eClinicalMedicine. 2023 Dec 21;67:102328. doi: 10.1016/j.eclinm.2023.102328 (PMC10776414; doi:10.1016/j.eclinm.2023.102328)
Supplement: Supplementary Material [file mmc1.pdf]

Supplementary Table 1: Number of mental disorders DALYs, with 95% UIs for Kenya, Both Sexes, Males, and Females, Ages 10-24, disaggregated by Risk

Population	Disorder	Risk Factor	Number [95% C.I]
	**Level 2 cause**		
Male	Mental disorders	Bullying victimization	12018.6 [4013.7; 23143.9]
Female	Mental disorders	Bullying victimization	15730.9 [5228.8; 30141.9]
**Both**	**Mental disorders**	**Bullying victimization**	**27749.5 [9280.1; 53549.5]**
Male	Mental disorders	Childhood sexual abuse	1821.0 [618.6; 3844.2]
Female	Mental disorders	Childhood sexual abuse	3964.3 [1287.5; 8538.0]
**Both**	**Mental disorders**	**Childhood sexual abuse**	**5785.2 [2256.5; 11180.9]**
Male	Mental disorders	Lead exposure	1983.9 [737.4; 3806.2]
Female	Mental disorders	Lead exposure	1548.1 [596.9; 2929.0]
**Both**	**Mental disorders**	**Lead exposure**	**3532.1 [1333.2; 6733.9]**
Male	Mental disorders	Intimate partner violence	0.0 [0.0; 0.0]
Female	Mental disorders	Intimate partner violence	4669.1 [15.4; 12118.6]
**Both**	**Mental disorders**	**Intimate partner violence**	**4669.1 [15.4; 12118.6]**
	**Level 3 cause**		
Male	Depressive disorders	Intimate partner violence	0.0 [0.0; 0.0]
Female	Depressive disorders	Intimate partner violence	4669.1 [15.4; 12118.6]
**Both**	**Depressive disorders**	**Intimate partner violence**	**4669.1 [15.4; 12118.6]**
Male	Depressive disorder	Bullying victimization	5906.6 [1656.5; 12465.9]
Female	Depressive disorder	Bullying victimization	7717.8 [2285.4; 16035.0]
**Both**	**Depressive disorder**	**Bullying victimization**	**13624.4 [4011.7; 28394.5]**
Male	Depressive disorder	Childhood sexual abuse	1821.0 [618.6; 3844.2]
Female	Depressive disorder	Childhood sexual abuse	3964.3 [1287.5; 8538.0]
**Both**	**Depressive disorder**	**Childhood sexual abuse**	**5785.2 [2256.5; 11180.9]**
Male	Anxiety disorders	Bullying victimization	6112.0 [2146.3; 12045.7]
Female	Anxiety disorders	Bullying victimization	8013.1 [2956.7; 15953.4]
**Both**	**Anxiety disorders**	**Bullying victimization**	**14125.1 [5029.9; 28264.3]**
Male	Idiopathic developmental intellectual disability	Lead exposure	1983.9 [737.4; 3806.2]
Female	Idiopathic developmental intellectual disability	Lead exposure	1548.1 [596.9; 2929.0]
**Both**	**Idiopathic developmental intellectual disability**	**Lead exposure**	**3532.1 [1333.2; 6733.9]**

Supplementary Table 2: Substance use disorders disaggregated by Risk Factors

**Population**	**Disorder**	**Risk Factor**	**Number [95% C.I]**
	Level 2 cause		
Male	Substance use disorders	Drug use	4906.5 [3271.1; 6943.9]
Female	Substance use disorders	Drug use	3563.2 [2188.4; 5502.9]
**Both**	**Substance use disorders**	**Drug use**	**8469.7 [5416.6; 12371.9]**
Male	Substance use disorders	Alcohol Use	3633.6 [2239.5; 5609.3]
Female	Substance use disorders	Alcohol Use	2919.1 [1775.5; 4557.1]
**Both**	**Substance use disorders**	**Alcohol Use**	**6552.7 [4022.6; 10208.9]**
Male	Substance use disorders	Childhood sexual abuse	504.0 [60.1; 1305.7]
Female	Substance use disorders	Childhood sexual abuse	556.4 [68.6; 1450.8]
**Both**	**Substance use disorders**	**Childhood sexual abuse**	**1060.4 [139.4; 2740.1]**

Supplementary Figure 1. Sex and age groups disaggregated DALYs for mental disorders

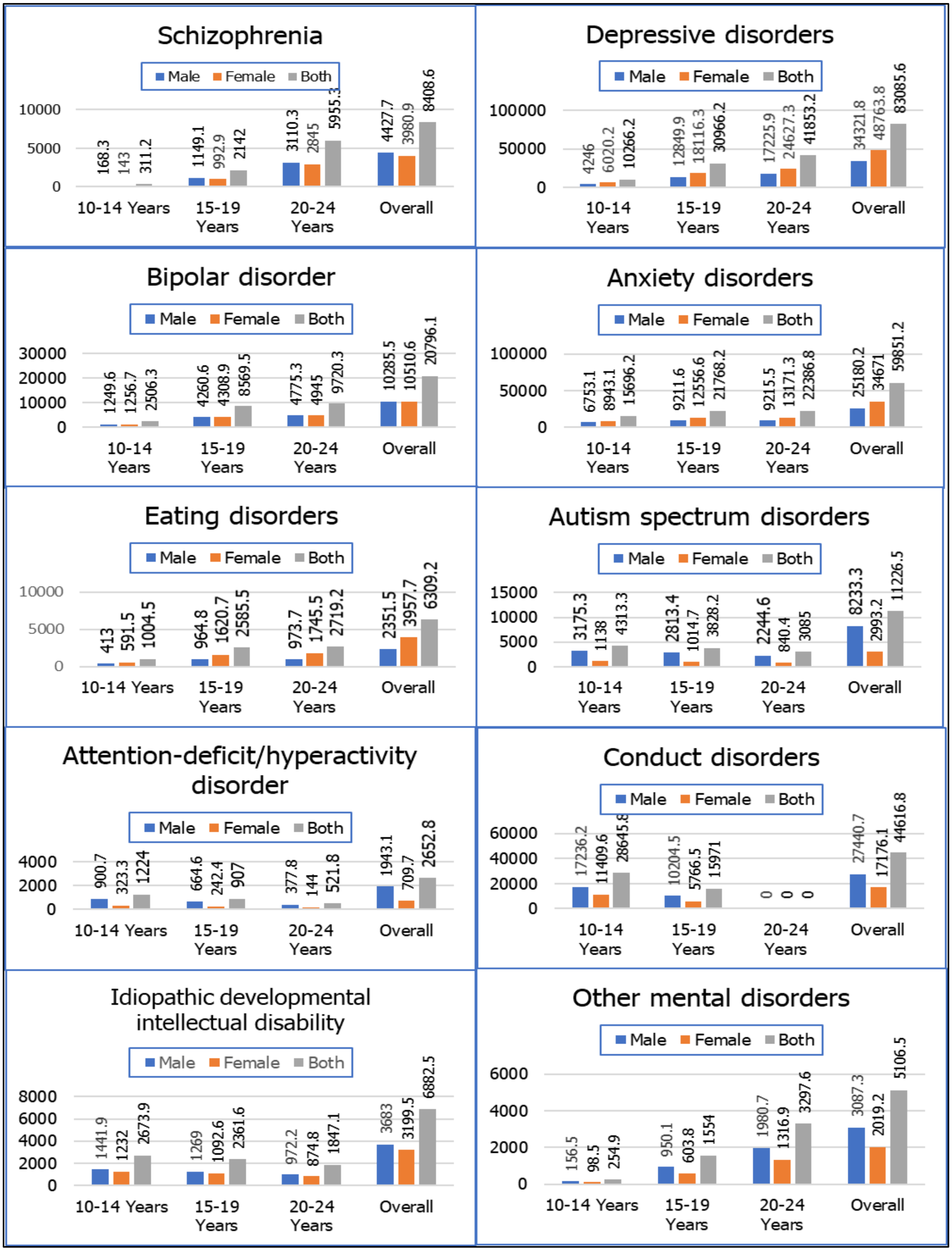

Supplementary Figure 2: Sex and age groups disaggregated data for substance use disorders

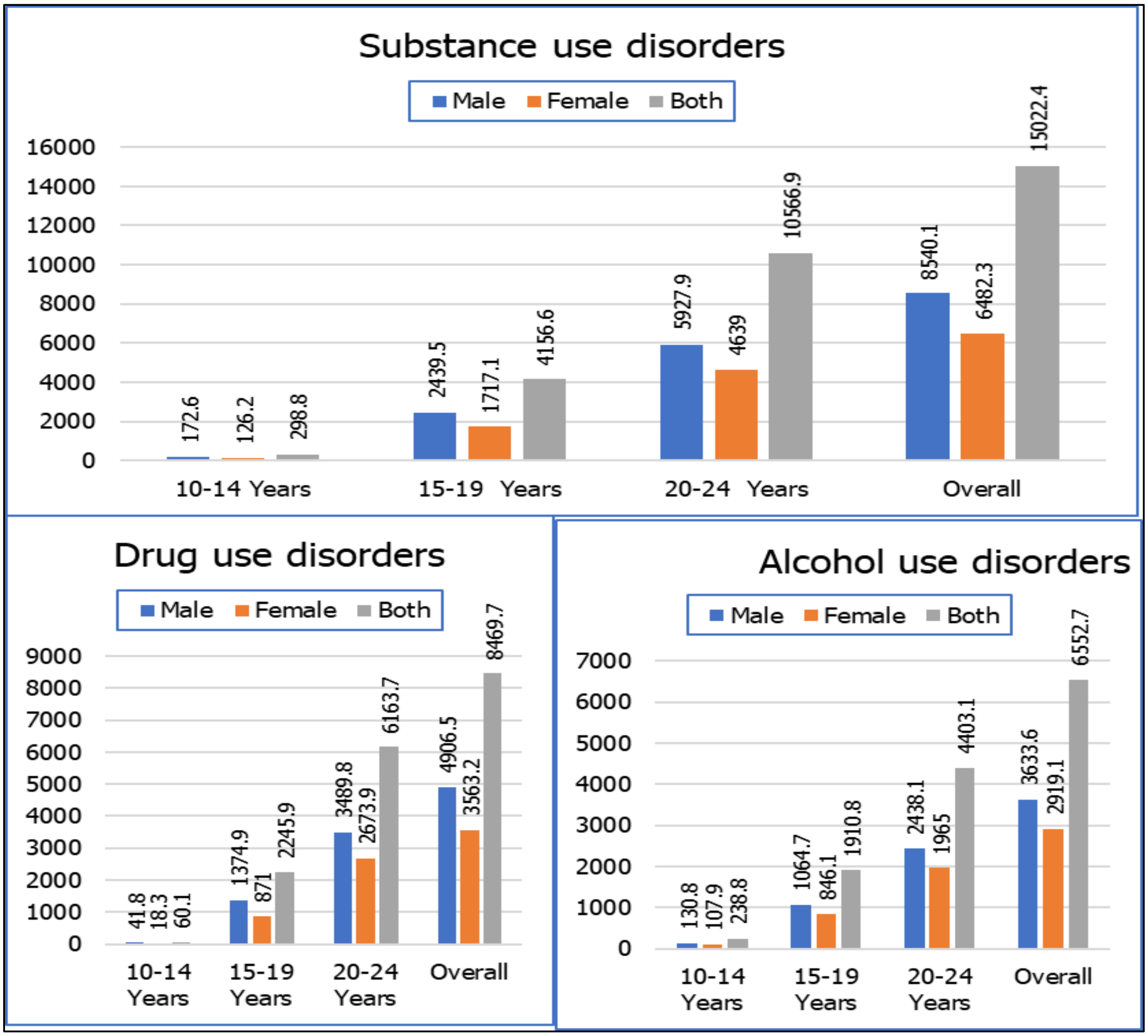

Appendix I: Authors Contributions

Providing data or critical feedback on data sources
Dickson A Amugsi, Manasi Kumar, Aggrey Gisiora Mokaya, Josephine W Ngunjiri, Albert Kimtai Tele, and Marcia Weaver

Developing methods or computational machinery
Josephine W Ngunjiri, Albert Kimtai Tele, and Marcia Weaver

Providing critical feedback on methods or results
Nabila Amin Ali, Dickson A Amugsi, David E O Bukusi, Simon Njuguna Kahonge, Mercy Wacera Karanja, Sarah Kanana Kiburi, Manasi Kumar, Aggrey Gisiora Mokaya, Josephine W Ngunjiri, Julius Nyerere Odhiambo, Nasri Abdullahi Omar, Albert Kimtai Tele, Richard G Wamai, Christine P M Wambugu, Mary Njeri Wanjau, Marcia Weaver, and Obadia K Yator

Drafting the work or revising it critically for important intellectual content
Dickson A Amugsi, Manasi Kumar, Josephine W Ngunjiri, Albert Kimtai Tele, Richard G Wamai, and Marcia Weaver

Managing the estimation or publications process
Manasi Kumar
